# Editorial for Special Issue “Antimicrobial Therapy in Intensive Care Unit”

**DOI:** 10.3390/antibiotics12020278

**Published:** 2023-01-31

**Authors:** Elizabeth Paramythiotou, Christina Routsi

**Affiliations:** 1 2nd Department of Intensive Care, School of Medicine, National and Kapodistrian University of Athens, ‘Attikon’ Hospital, 12462 Athens, Greece; 2 1st Department of Intensive Care, School of Medicine, National and Kapodistrian University of Athens, ICU “Evangelismos” Hospital, 10676 Athens, Greece

Life-threatening infections, either as the initial reason for an admission to the intensive care unit (ICU) or acquired in the ICU, are especially common among critically ill patients. As a result, patients hospitalized in the ICU have a great exposure to multiple antimicrobial and antifungal agents. Antimicrobial therapy in the ICU has been challenging due to the emergence and the increasing incidence of difficult-to-treat and multidrug-resistant pathogens. Furthermore, during the ongoing pandemic, the number of patients who are hospitalized in the ICU due to COVID-19 has greatly increased with the concomitant increase in antimicrobial exposure. In addition, organ support techniques, including renal replacement therapy and extracorporeal membrane oxygenation (ECMO), further complicate the appropriate antimicrobial treatment in terms of dosing and the way the drug is administered. 

The current special edition of *Antibiotics* entitled "Antimicrobial Therapy in Intensive Care Unit” brings together 15 important articles which are presenting the current evidence on the antimicrobial treatment in the ICU and the associated issues. It includes seven original articles and eight comprehensive reviews dealing with a great diversity of subjects and the factors affecting the outcome of the frail and the often subdued to long treatments in the ICU. 

This Special Issue begins with an excellent review article by Tabah et al. [1] on the antimicrobial management of bloodstream infections focusing on the importance of microbiology specimens, the timing and choice of the empirical antimicrobial therapy, the role of spectrum and dose optimization, the importance of source control, and, finally, strategies for stopping antimicrobials. 

Next, Karaiskos and Giamarellou [2] place emphasis on the difficult-to-treat and pandrug-resistant Gram-negative bacteria in critically ill patients by reviewing salvage antibiotics treatments, synergistic combinations, as well as an increased exposure regimen adapted to the MIC of the pathogen. Furthermore, this review article contains a report on novel antimicrobial agents, namely the lactam-beta-lactamase inhibitor combinations cefiderocol and eravacycline.

In their systematic review, Karakonstantis and colleagues [3] summarize well the currently available approaches to the management of pandrug-resistant *Acinetobacter baumannii.* The authors propose antimicrobial combinations which have been guided by an in vitro synergy evaluation as the most appropriate treatment option. 

Excess antibiotic use is one of the factors contributing to the emergence of bacterial resistance. Therefore, the de-escalation of empirical regimens is a principal component of antimicrobial stewardship programs. Cumulative evidence supporting the use of procalcitonin guidance in promoting antimicrobial stewardship for critically ill patients by the restriction of an injudicious antimicrobial treatment has been presented by Kyriazopoulou and Giamarellos-Bourboulis in their review article [4]. The authors conclude that according to the current evidence biomarkers, mainly procalcitonin should be implemented in antimicrobial stewardship programs, including also the COVID-19 pandemic.

In their nice study, Rizk et al. [5] describe the impact of combining antimicrobial stewardship and infection control measures on resistance rates and colonization pressure of carbapenem-resistant *Acinetobacter baummanni* (CRAb) in the ICUs of a tertiary care center in Lebanon before the COVID-19 pandemic. They demonstrate that a multidisciplinary approach and combined interventions between the stewardship and infection control teams can lead to a sustained reduction in resistance rates and the spread of CRAb in ICUs.

The article by Routsi et al. [6] confirms that the incidence of candidemia in the ICU has increased in COVID-19 patients compared to the pre-pandemic era and it highlights the marked increase in the resistance to fluconazole as well as the emergence of *C. auris.*

Lau et al. [7] provides important information regarding the utilization of antibiotics in the South East Asia region. In a retrospective study over the past six years, the authors recorded antibiotics and specifically the consumption of carbapenem in the general and in the COVID-19 ICUs of a Malaysian hospital. They found that the consumption of antibiotics increased markedly in the year 2021 compared to previous years. The excessive consumption of antibiotics was partially attributed to an unwarranted empirical use over a prolonged period and to the infrequent application of antimicrobial de-escalation.

There are two separate papers that examined ICU-acquired blood stream infections. In the first one is by Mantzarlis et al. [8]. The investigators examined secondary infections in patients admitted to the ICU due to COVID-19 over a period of 9 months. They demonstrated a high incidence of 57% of blood stream infections. Multidrug-resistant *Acinetobacter baumannii* and *Klebsiella pneumoniae* were the most common isolated pathogens. However, in the multivariate analysis, the illness severity on ICU admission was the only independent risk factor for mortality. The second paper by Karvouniaris et al. [9] examined retrospectively the impact of ICU-acquired Gram-negative blood stream infections on mortality in a regional Greek hospital. Patients with blood stream infections due to colistin-resistant strains were compared to those with colistin-sensitive strains. The authors demonstrate that the sepsis severity was the independent predictor of mortality regardless of the colistin-resistance phenotype or empirical colistin treatment.

Two studies published in this issue of *Antibiotics* address ventilator-associated pneumonia (VAP). Given the global increase in antibiotic resistance, particularly among Gram-negative bacilli and the difficulty in choosing empiric therapy, Chaibi et al. [10], in their review article, present the difficulties in the management of VAP. The empiric use of newly available antibiotics is discussed along with the presentation of the current epidemiological data in terms of multidrug-resistant pathogens, as well as the clinical and microbiological elements that should be considered when an empirical therapy is started. In the same context, Adukauskiene et al. [11], in their research article, have investigated the clinical features and the 30-day mortality of VAP due to multidrug-resistant *A. baumannii* (MDRAB) in a reference Lithuanian university hospital. Both monobacterial and polybacterial MDRAB VAP episodes during a two-year period were retrospectively studied. It was demonstrated that monobacterial MDRAB VAP had different demographic/clinical characteristics compared to polybacterial and carried worse outcomes. 

One of the main problems in treating infections in critically ill patients is the difficulty to achieve the pharmacodynamic targets. This Special Issue offers three articles addressing this topic. Extracorporeal membrane oxygenation (ECMO), a temporary mechanical cardiorespiratory support, is a relatively new development increasingly used in modern ICU as a bridge to recovery in otherwise irrecoverable patients. Both critical illness and ECMO alter the pharmacokinetics (PK) and pharmacodynamics (PD) of administered drugs and challenge appropriate antibiotic regiments. The review by Gomez et al. [12] thoroughly summarizes PK/PD alterations in critically ill patients receiving ECMO, emphasizing the practical application and reviewing patient-, illness-, and ECMO hardware-related factors. Jang and colleagues [13] have provided an interesting analysis to determine whether a patient’s weight influences the probability of target attainment (PTA) over 72 h of initial therapy with beta-lactam and carbapenem antibiotics in critical care patients under continuous renal replacement therapy. By using Monte Carlo simulations, it was shown that patients in lower weight quartiles tended to achieve higher antibiotic pharmacodynamic target attainment compared to heavier patients. In addition, in the context of the increasing incidence of multidrug resistance, Dhaese et al. [14] in a great perspective article suggest the new concept of the maximum tolerable dose (MTD. MTD has been defined as the highest dose of an antimicrobial drug deemed safe for the patient. Maximizing the death of bacterial cells and minimizing the risk of antimicrobial resistance and toxicity is the goal in the introduction of this concept. The authors provide a theoretical approach of how increasing uremic toxin concentrations could be used as a quantifiable marker of beta-lactam antibiotic toxicity, thus suggesting directions for future research. 

Finally, Schuurman et al. [15], in a thorough review, describe the gut microbiome in health and disease. The authors discuss the concept of a probiotic intervention to positively modulate the gut microbiome. They summarize the evidence from randomized clinical trials and focus on the prevention of ventilator-associated pneumonia.

We wish to thank all the authors for their comprehensive contributions to this Special Issue of *Antibiotics* and hope that the readers will find interest in the content.
